# Diarrhea Prevention Practice and Associated Factors among Caregivers of Under-Five Children in Enemay District, Northwest Ethiopia

**DOI:** 10.1155/2019/5490716

**Published:** 2019-05-12

**Authors:** Melese Dubie Agegnehu, Liknaw Bewket Zeleke, Yitayal Ayalew Goshu, Yonas Lamore Ortibo, Yohannes Mehretie Adinew

**Affiliations:** ^1^World Vision, Addis Ababa, Ethiopia; ^2^College of Health Sciences, Debre Markos University, Debre Markos, Ethiopia; ^3^College of Health Sciences and Medicine, Debre Tabor University, Debre Tabor, Ethiopia; ^4^College of Health Sciences and Medicine, Wolaita Sodo University, Ethiopia

## Abstract

**Background:**

Diarrhea is the leading cause of mortality among infants and children younger than 5 years of age in both underdeveloped and developing countries. Factors determining the occurrence of diarrhea in children are complex, and the relative contribution of each factor varies as a function of interaction between socioeconomic, environmental, and behavioral variables.

**Objectives:**

To assess diarrhea prevention practice and associated factors of diarrheal disease among caregivers who have under-five children in Enemay district, Ethiopia, 2018.

**Methods:**

Community-based cross-sectional study was done from June 1–30, 2018, among 398 caregivers who have under-five children, in the Enemay district that were selected by using the simple random sampling technique. A structured and pretested data collection tool was used to collect the data. Data were entered using EPI DATA version 4.2, and analysis was done using SPSS version 20 statistical package to be cleaned and analyzed. Descriptive analysis was done to describe study participants, and logistic regression (bivariable and multivariable) analysis was done to identify factors that have association with the dependent variable. The *P* value was less than 0.05.

**Results:**

A total of 398 with a response rate of 97% under-five caregivers were participated in this study. Nearly, half (48.7%) of the participants were in the age group 25–34. The study revealed that good practice of diarrhea prevention was 52.8%. This study was also identified that occupation (AOR: 3.922, 95% CI: 1.593, 9.657), family size (AOR: 0.088, 95% CI: 0.009, 0.916), and understanding on diarrhea (AOR: 0.237, 95% CI: 0.091, 0.613) were associated factors of diarrhea prevention practice of under-five children caregivers.

**Conclusion:**

This finding showed that diarrhea prevention practice among under-five children caregivers was low and prevention practice was significantly associated with caregivers' awareness on frequency of diarrhea in a day, occupation, and family size in a house.

## 1. Background

Diarrhea is the passage of loose or watery stools occurring three or more times in a 24-hour period which means an increased frequency or decreased consistency of bowel movements, and it affects people of all ages [1]. It is usually a symptom of an infection in the intestinal tract, which can be caused by a variety of bacterial, viral, and parasitic organisms [2].

Diarrhea is the second leading cause of child morbidity and mortality, especially in the developing countries. Globally, it is estimated that there are 2.5 billion episodes and 1.5 million deaths annually in children under five years [3].

Diarrheal diseases are major causes of malnutrition, delayed physical development, and early childhood mortality in developing countries and poor communities, and the major cause of death in children with diarrhea is loss of water and essential minerals [4].

In sub-Saharan Africa, primary caregivers display poor perception about the signs of dehydration, dysentery, and management of diarrhea [5].

The incidence of illnesses contributing to avoidable deaths diarrhea is higher in Ethiopia compared to other sub-Saharan African countries partly due to different factors [6]. In Ethiopia, diarrheal disease is a major public health problem, and it is one of the top 15 countries in which nearly three-fourths of child deaths occur due to diarrhea [4]. In Ethiopia, morbidity reports and community-based studies indicate that diarrheal diseases are a major public health problem that causes excess morbidity and mortality among children [7]. The prevalence in Enemay woreda is 18.6% which indicates diarrhea is still the burden in the study area [8].

Various preventive techniques were reported in the literatures including hygiene and sanitation, diet, medications, and supplements which are generally classified as health care, breastfeeding, immunization, supplemental zinc, and probiotics [9]. Treatment and prevention of diarrhea can be done at home by primary caregivers, and their role is vital in health promotion, disease prevention, and patient care [4].

Prevention practice of caregivers is important and can prevent diarrhea-related child morbidity and mortality. Therefore, the study aimed at identifying the gaps on under-five children diarrhea prevention practice and associated factors in the study area so as to forward recommendations for under-five caregivers, local health-care providers, and other stakeholders to reduce diarrhea-related morbidity and mortality. The community at large in the study area can be benefited from improved under-five diarrhea prevention practice.

## 2. Methods

### 2.1. Study Design

Community-based cross-sectional study was conducted.

### 2.2. Study Area and Period

The study was conducted in Enemay district, East Gojjam zone, Amhara regional state, northwest Ethiopia, from June 1–30, 2018. The Enemay district is located 370 km, northwest of Addis Ababa, the capital city of Ethiopia, and 220 km southwest of Bahir Dar city, the capital of the Amhara National Regional State, respectively. The total population in the district is 198241, of which 26404 are under-five children. The district has one district hospital, 7 health centers, and 34 health postsites. All these health facilities involve in diarrhea prevention and control. Access to safe water supply in the district is 62% [10].

### 2.3. Source Population

All caregivers of under-five children in the Enemay district were the source populations, and the study population was all caregivers of under-five children in the selected kebeles.

### 2.4. Sample Size Determination

Single population proportion formula was employed using population proportion 41.3% [5].

With confidence level 95% and margin of error 5%, *n*=(*Z*_*α*_/2)^2^*p*(1 − *P*)/*d*^2^, where *n* is the sample size, *P* is the population proportion of diarrhea prevalence, *d* is the margin of error (0.05), and *α* is 5%.

So using the above formula, the sample size is *n*=*z*^2^*p*((1 − *p*)/*d*^2^)=((1.96)^2^ × 0.413)((1 − 0.413)/(0.05)^2^)=373.

The final sample size with 10% nonresponse rate is 410.

### 2.5. Sampling Technique

The study was conducted using simple random sampling. Six kebeles were selected by lottery method from all 29 kebeles in the Enemay district. Finally, the required number of under-five caregivers was selected by using the simple random sampling technique from the list obtained from health extension workers in selected kebeles.

### 2.6. Data Collection Procedure

Data were collected by interviewer-administered interview and observation using semistructured questionnaire. The questionnaire was prepared in English initially by reviewing the literatures and then translated in to the Amharic version which later on translated back to the English version to check its consistency and comparability of the finding. Six health extension data collectors and one BSc nursing supervisor were recruited to data collection in the Enemay district. Training was given for data collectors and supervisor on objectives of the study, method of collecting the needed information through interviewing and observation, how to fill the information on the questionnaire, and the ethical aspect in approaching the participants which should be in a polite and respectful manner. The supervisor had been monitoring the data collection process, and the investigators were available to respond the concerns raised from the data collector and the supervisor. The older children were considered when two or more under-five children were found in the house.

### 2.7. Operational Definition

#### 2.7.1. Good Diarrhea Prevention Practice

Participants who scored above the mean value of practice question [11].

#### 2.7.2. Clean Latrine

No fecal matter in and around the pit latrine, properly swept.

#### 2.7.3. Hand Washing at Critical Time

It includes washing hand after using a toilet, after cleaning a child, after any cleaning activity, before preparing food, before meal, and before breastfeeding.

#### 2.7.4. Caregiver

A caregiver is an individual who could be a parent, foster parent, or head of the household who attends to the needs of a child or primary caregiver [12].

### 2.8. Data Quality Assurance

The questionnaire was adapted from literatures and modified into local context. Training was given for data collectors and supervisor. Pretesting of the questionnaire was made on 5% of the sample size in adjacent kebele prior to the actual data collection. The data collection process was strictly followed day to day by the supervisor, and data were checked for its completeness by the principal investigator. During analysis, not eligible variables for the analysis method were excluded by checking the chi-square test, and the model fitness test was also computed.

### 2.9. Data Analysis Technique

Data were coded and entered into EPI DATA version 4.2 and exported to SPSS version 20 for statistical analysis. Descriptive analysis was done to describe study participants in terms sociodemographic characteristics. Bivariate logistic regression analysis was done to determine the association between each independent and dependent variable. All factors that were associated with the dependent variable in bivariate analysis with a *P* value of 0.20 or less were included in the beginning of multivariable analysis to identify the associated variables. A *P* value < 0.05 and corresponding 95% CI of odds ratio were considered to declare a result as statistically significant in this study in multivariable regression. The odds ratio together with 95% confidence intervals was used to interpret the strength and direction of association.

### 2.10. Ethical Consideration

Ethical clearance was obtained from Ethical Review Committee, College of Health Sciences, Debre Markos University, and letter of permission was obtained from the Enemay district health office. The purpose of the study was explained to respondents, and verbal informed consent was obtained from participants. Confidentiality of information was maintained by keeping anonymous personal identifiers. Children who were found with active diarrheal disease during house-to-house visit for data collection were linked to health facilities for treatment.

## 3. Results

### 3.1. Sociodemographic Characteristics

A total of 398 under-five children caregivers participated in the study, making the response rate of the study as 97%. Among the respondents, nearly half (48.7%) were between 25–34 years with the mean age of 33.2. Around two-thirds of the participants (66.1%) and their husband (65.1%) had no formal education. Concerning occupation of the participants, majority (87.2%) participants were housewives. Regarding family size, 228 (57.3%) participants had less than four family members per household and 392 (98.5%) of caregivers had less than two under-five children. (Table 1)

### 3.2. Knowledge on Diarrhea Prevention Practice

Out of 398 caregivers, 62.6% had information on diarrhea and nearly one-third (34.4%) of the participants recognized diarrhea as passage of watery stool once in a day. More than half of the caregivers considered latrine utilization (54.8%) and hand washing (51.5%) as prevention methods for diarrhea. In addition, 261 (65.6%) of caregivers respond watery diarrhea as sign and symptoms of diarrhea. Questions were also asked for caregivers about causes of diarrhea, and 70.1% respond contaminated food. Concerning treatment seeking behavior of the participants, 37 (31.9%) participants stay at home without treatment when their child had diarrhea (Table 2).

### 3.3. Attitude towards Diarrhea Prevention Practice

About 252 (60.6%) participants disagreed on the likelihood of their child to get diarrhea in the next month whereas one hundred fifty eight (37.7%) of caregivers disagreed that diarrhea is normal in children. About 166 (39.2%) of caregivers agreed that diarrhea is a preventable disease, and 110 (27.6%) of caregivers disagreed that diarrhea is a preventable disease. Among the total participants, nearly half (50.5%) agreed that diarrhea is a communicable disease and 41.5% of caregivers strongly agreed that diarrhea can be caused by open defecation.

### 3.4. Environmental Factors

From the total study participants, nearly two-thirds (68.8%) had latrine in their premise from which almost half (50.4%) were unimproved. Concerning hand washing facility, 147 (36.9%) participants had hand washing facility in their premise during study period. Nearly one out of five participants (80.2%) of caregivers obtain drinking water from well-protected source and around half (55%) of caregivers spend longer than 30 minutes (round trip) to obtain their drinking water.

### 3.5. Under-Five Caregivers' Diarrhea Prevention Practice

Two hundred forty nine (62.6%) of the participants treat their drinking water source using chlorine, and 233 (58.4%) of participants use latrine for defecation. From the total study participants, under-five caregivers asked about hand washing times, 272 (68.3%) respond that they wash their hands after toilet visit and only 151 (37.9%) wash their hands before child feeding. One hundred sixty four (41.2%) caregivers wash their hands using only water. Only 27% latrines were clean during data collection. About half (50.5%) of caregivers feed their child exclusively for six months (Table 3).

As shown by the figure below, the overall diarrhea prevention practice among caregivers' of under-five children in the Enemay district, Amhara regional state, northwest Ethiopia, was 52.8%.

### 3.6. Factors Associated with Diarrhea Prevention Practice

Factors associated with diarrhea prevention were identified by computing bivariate and multivariate analyses. Occupation, knowledge on immunization to prevent diarrhea, family size, information on diarrhea, and fluid importance during diarrhea sickness and awareness of diarrhea frequency showed association in bivariate analysis whereas in multivariable analysis, occupation of caregivers, family size, and awareness on diarrhea frequency showed significant association.

Under-five diarrhea prevention practice among housewives was 3.9 times more likely (AOR: 3.922, 95% CI: 1.593, 9.656) than merchants and others.

Diarrhea prevention practice in caregivers with family size 5–8 was 91.2% less likely (AOR: 0.088, 95% CI: 0.009, 0.916) than those that have family size less four.

Caregivers who perceived diarrhea as passage of watery stool two times in a day were 76.3% less likely (0.091, 0.613) to practice diarrhea prevention in under-five children than who perceived three times in a day (Table 4).

## 4. Discussion

From the total participants of the study, 58.4% were utilizing latrine properly. The findings are in line with the study in Gulomekada District, north Ethiopia, where rate of latrine utilization was about 57.3% [13]. This finding is higher than a study finding reported from Farta Woreda, northwest Ethiopia, where latrine utilization habit was 29.2%, and Asmara, Eritrea, which indicated 72.2% of participants are defecating in open field [6, 14]. The difference might be attributed to the difference in the sociodemographic characteristics and basic environmental infrastructure of study households, behaviors of the participants, and awareness of the community.

The present study revealed that 62.6% of caregivers use chlorine-treated drinking water. This is higher than a study finding in Farta Woreda, northwest Ethiopia, where only 4.9% of the participants treat their drinking water and lower than a finding in Dejene woreda, Ethiopia, where 81% add bleach to treat drinking water [6, 15]. The difference might be due to sociodemographic factors, accessibility of supplies, and difference in study period.

In this study, 41.2% of caregivers wash their hands using only water which may not be effective in removing of disease-causing bacteria, and it could be source of contamination during washing their hand which is another risk for diarrhea transmission. The reported practice of hand washing is slightly higher than the observed availability of hand washing facility. The disparity may be resulted from utilization of traditional methods of hand washing without preparing or availing hand washing facilities. In the other case, hand washing with soap was lower (26.4%) than with plain water. The discrepancy might be due to unaffordability for frequent buying of soap. Similar finding was reported by a study carried out in India that demonstrated 41% hand washing is with using water only [16]. This finding is less than the study conducted in Farta, Ethiopia, where 56.3% of the respondents used only water to wash their hands [6]. The difference might be due to sociodemographic and economic factors.

In this study, caregivers were asked about hand washing at critical times, and from the total participants, 65.1% wash their hands before meal, 50% wash their hands before food preparation, 37.9% wash their hands before feeding children, 9.8% wash their hands after cleaning children, 50.3% wash their hands after any cleaning activity, and 68.3% wash their hands after visiting toilet.

The present study revealed that about 73% latrines were clean. This is line with the study conducted in Aneded District, northwest Ethiopia, where about 66.7% of latrines were clean [17]. This might be due to similar sociodemographic status of the community.

The findings of this study revealed that 63.1% of caregivers practiced exclusive breastfeeding for six months. This finding is different from that of the study conducted in Jigjiga District, Somali Region, eastern Ethiopia in which 84.4% of the children were not exclusively breastfed in the first 6 months of their life and in 33.6% in Ibadan, Nigeria [7, 18]. The difference might be due to sociodemographic factors and difference in study design.

This study also found that about 88.9% of children completed their course of vaccination. This study is different from a study conducted in Dejen district, northwest Ethiopia, where only 48.6% were fully vaccinated [15]. The difference might be due difference level of awareness, accessibility of health facility, and supplies.

In this finding, about 52.8% of caregivers scored above the mean value of practice-related questions that had good diarrhea prevention practice. This is lower than from a study conducted in Indonesia where 68.3% of caregivers had good behaviors of preventing diarrhea [19]. The difference might be due to level of awareness, educational status, sociodemographic factors, and others. The finding is higher than findings of studies conducted in Fagita Lekoma district (37.6%) [19], South Sudan (42.2%) [20], and Finote Selam town (45.9%) [6].

In this study, housewife caregivers were 3.9 times more likely to practice diarrhea prevention in under-five children than merchants and others (AOR: 3.922, 95% CI: 1.593, 9.657). This is supported by the study done in Iran, where occupation was significantly associated with diarrhea prevention practice [21]. The reason may be that housewives could have the opportunity to get information from different sources as they have sufficient time to gain information from different sources and to practice it. This finding is different from the study conducted in Fagita Lekoma district, Ethiopia, where occupation was not significantly associated with diarrhea prevention [22]. This difference might be due to level of education, sociodemographic status, and sociocultural characteristics.

In this study, caregivers who had 5 or more family size were 91.2% less likely to practice diarrhea prevention in under-five children than those who had less than four family size. This finding is in agreement with a study finding from Gojam Hullet Ejju Ense woreda, Ethiopia, where greater family size was associated with diarrhea morbidity [23]. But contradicts from a study finding in Derashe District, Southern Ethiopia, where family size was not significantly associated with diarrhea disease [20]. The difference might be socioeconomic status, environmental factors, education level, living condition, and difference in time of study.

Caregivers who respond frequency of diarrhea two times were 76.3% less likely to practice than who answered three times in a day. This is the finding supported by the study conducted in Ethiopia where mothers who had no understanding about diarrhea were by 80.3% less likely to have good practice compared with their counterparts [22]. This may be due to the fact that mothers who had information about diarrhea have a good opportunity to good prevention practice. This study is different from a study conducted in Nigeria, where 93% respondents were aware of diarrhea and had understanding on it [24]. This might be due to caregivers' lack of prior experience, educational status, and source of information.

The key strengths of the study were the design which is community based and involvement of adequate sample size. The basic limitations of the study were difficulty to know the cause and effect, at the same time resulted from its nature of cross-sectional study and under or over reported depending on the recent health-care workers activity because they might think of that children could receive medical attention through the study.

## 5. Conclusion

The result of this study showed that diarrhea prevention practice among under-five children caregivers was low, and the practice of diarrhea prevention practice was significantly associated with information on frequency of diarrhea, occupation, and family size in a house. Stakeholders have to focus on efforts to control diarrheal diseases and on increasing the level of knowledge and behavior of diarrhea prevention practice through increasing family planning and behavioral change.

## Figures and Tables

**Table 1:** Distribution of sociodemographic characteristics of study caregivers' in Enemay district, northwest Ethiopia, 2018.

Variable name	Category	Frequency	Percent
Age	15–24	50	12.6
	25–34	194	48.7
	>35	154	38.7

Sex	Male	8	2.0
	Female	390	98.0

Religion	Orthodox	294	73.9
	Muslim	103	26.1

Occupation	Housewife	347	87.2
	Student	7	1.8
	Employed	11	2.8
	Merchant	33	8.3

Education	No formal education	263	66.1
	Primary (1–8)	111	27.9
	Secondary (9–12) and above	24	6.0

Husband education	No formal education	259	65.1
	Primary (1–8)	119	29.9
	Secondary (9–12) and above	20	5.0

Family size	<4	228	57.3
	5–8	170	42.7

No. of 5 < children	1-2	392	98.5
	3-4	6	1.5

Relation to child	Mother	301	75.6
	Sister	89	22.4
	Father	8	2

Average monthly income	1401–2350	215	54
	2351–5000	131	33
	>5000	5	1.2
	No income	47	11.8

Marital status	Single	11	2.8
	Married	382	95.9
	Divorced	5	1.3

**Table 2:** Knowledge distribution of caregivers about under-five diarrhea prevention in Enemay district, northwest Ethiopia, 2018.

Variable name	Category	Frequency	Percent
Information on diarrhea	Yes	249	62.6
	No	149	37.4

Awareness on frequency of diarrhea	Watery diarrhea once in a day	137	34.4
	Watery diarrhea twice in day	95	23.9
	Watery diarrhea three times in a day	78	19.6
	Watery diarrhea four times in a day	88	22.1

Sign and symptom of diarrhea	Vomiting	118	29.6
	Fever	130	32.7
	Dehydration	163	40.9
	Appetite loss	176	44.2
	Weakness	144	36.2
	Watery diarrhea	261	65.6

Cause of diarrhea	Insects	99	24.9
	Contaminated water	262	65.8
	Contaminated food	279	70.1
	Bad sprit	76	19.1
	Poor sanitation	197	49.5
	Bad smell	64	16.1

Type of diarrhea you know	Bloody	116	29.1
	Watery with no blood	282	70.9

Diarrhea treatment	No treatment	37	31.9
	Homemade ORS	33	28.4
	Took to health facility	36	31.1
	Visit traditional healer and others	10	8.6

Prevention of diarrhea	Breastfeeding	173	43.5
	Eat safe food	202	50.8
	Use latrine	218	54.8
	Immunization	182	45.7
	Hand washing	205	51.5

**Table 3:** Under-five caregivers' diarrhea prevention practice in Enemay district, northwest Ethiopia, 2018.

Variable name	Category	Frequency	Percent
Water treatment	Boiling	95	23.9
	Chlorine	249	62.6
	Filter with close	14	3.5
	Nothing	40	10.1
	After toilet visit	272	68.3

Hand washing	Before food preparation	199	50
	Before meal	259	65.1
	After any cleaning	212	50.3
	Before feeding/breastfeeding	151	37.9
	After child cleaning	139	9.8

Hand hygiene materials	Water only	164	41.2
	Water with ash	129	32.4
	Water with soap	105	26.4

Was the latrine clean?	Yes	200	73.0
	No	74	27.0

Exclusive breastfeeding	>6 month	127	31.9
	6 month	251	63.1
	<6 month	20	5.0

Immunization status	Completed	354	88.9
	Not completed	44	11.1

Initiation of first breast milk	Within 1 hour	282	70.8
	After 1 hour	116	29.2

**Table 4:** Factors associated with under-five diarrhea prevention practice among caregivers, Enemay district, Ethiopia, 2018.

Variables		Prevention practice		COR (95% CI)	AOR (95% CI)
		Poor	Good		
Immunization	Yes	91	91	1.23 (0.49, 0.91)	1.17 (0.71, 1.94)
Prevent diarrhea	No	6 97	119	1	1
Occupation	House wife	168	179	1.27 (1.01, 1.59)	3.92 (1.59, 9.66)^*∗*^
	Merchants and others	20	31	1	1
Family size	≥5	61	109	0.45 (1.33, 2.89)	0.09 (0.01, 0.92)^*∗*^
	≤4	127	101	1	1
Information on diarrhea	Yes	127	122	0.67 (0.44, 0.99)	0.1.503 (0.86, 2.64)
	No	61	88	1	1
Frequency of diarrhea	Once/twice/day	96	136	0.64 (1.61, 7.67)	0.24 (0.09, 0.61)^*∗*^
	>/ = times/day	87	79	1	1
Fluid during diarrhea	Yes	111	114	1.71 (1.15, 2.55)	1.111 (0.65,1.90)
	No	77	96	1	1

^*∗*^Significantly associated; COR, crude odds ratio; AOR, adjusted odds ratio.

## Data Availability

The data used to support the findings of this study are available from the corresponding author upon request.
